# EIF4A3‐Induced CircDHTKD1 regulates glycolysis in non‐small cell lung cancer via stabilizing PFKL

**DOI:** 10.1111/jcmm.18465

**Published:** 2024-07-18

**Authors:** Zhenghua Liu, Wenya Li, Ziyi Wang, Qiwei Yang, Liang Chen, Weiyang Chen, Xiaohan Qu

**Affiliations:** ^1^ Department of Thoracic Surgery The First Hospital of China Medical University Shenyang Liaoning China

**Keywords:** CircDHTKD1, EIF4A3, glycolysis, IGF2BP2, PFKL

## Abstract

Lung cancer (LC) is one of the malignancies with the highest incidence and mortality in the world, approximately 85% of which is non‐small cell lung cancer (NSCLC). Circular RNAs (circRNAs) exert multiple roles in NSCLC occurrence and development. The sequencing results in previous literature have illustrated that multiple circRNAs exhibit upregulation in NSCLC. We attempted to figure out which circRNA exerts an oncogenic role in NSLCL progression. RT‐qPCR evaluated circDHTKD1 level in NSCLC tissue specimens and cells. Reverse transcription as well as RNase R digestion assay evaluated circDHTKD1 circular characterization in NSCLC cells. FISH determined circDHTKD1 subcellular distribution in NSCLC cells. Loss‐ and gain‐of‐function assays clarified circDHTKD1 role in NSCLC cell growth, tumour growth and glycolysis. Bioinformatics and RIP and RNA pull‐down assessed association of circDHTKD1 with upstream molecule Eukaryotic initiation factor 4A‐III (EIF4A3) or downstream molecule phosphofructokinase‐1 liver type (PFKL) and insulin‐like growth factor 2 mRNA binding protein 2 (IGF2BP2) in NSCLC cells. Rescue assays assessed regulatory function of PFKL in circDHTKD1‐meidated NSCLC cellular phenotypes. CircDHTKD1 exhibited upregulation and stable circular nature in NSCLC cells. EIF4A3 upregulated circDHTKD1 in NSCLC cells. CircDHTKD1 exerted a promoting influence on NSCLC cell malignant phenotypes and tumour growth. CircDHTKD1 exerted a promoting influence on NSCLC glucose metabolism. CircDHTKD1 exerts a promoting influence on NSCLC glucose metabolism through PFKL upregulation. RIP and RNA pull‐down showed that circDHTKD1 could bind to IGF2BP, PFKL could bind to IGF2BP2, and circDHTKD1 promoted the binding of PFKL to IGF2BP2. In addition, RT‐qPCR showed that IGF2BP2 knockdown promoted PFKL mRNA degradation, suggesting that IGF2BP2 stabilized PFKL in NSCLC cells. CircDHTKD1 exhibits upregulation in NSCLC. We innovatively validate that EIF4A3‐triggered circDHTKD1 upregulation facilitates NSCLC glycolysis through recruiting m6A reader IGF2BP2 to stabilize PFKL, which may provide a new direction for seeking targeted therapy plans of NSCLC.

## 1 INTRODUCTION

Lung cancer (LC) is one of the malignancies with the highest incidence and mortality in China and even in the world.^1^ Approximately 85% of LC is non‐small cell lung cancer (NSCLC).^2^ The 5‐year survival rate of early NSCLC after surgical therapy can reach 70%–90%,^3^ whereas approximately 75% of NSCLC patients are in progressive or advanced stages at the time of initial diagnosis.^4^ Thus, seeking NSCLC biomarkers and therapeutic targets with good specificity and sensitivity has become focus and difficulty of prolonging survival and elevating quality of life of NSCLC patients.

In 2011, *Cell* published a review article, attributing abnormal metabolic changes to 1 of the 10 typical characteristics of tumour cells.^5^ During growth of solid tumours such as NSCLC, rapid cancer cell proliferation results in cancer tissue microenvironment to remain in a relatively low‐oxygen state.^6^ Hypoxia can cause changes in gene and protein expression in tumour cells, resulting in tumour growth restriction, cell cycle arrest, apoptosis and necrosis; simultaneously, hypoxia can also repress apoptosis through triggering angiogenesis, enhancing glycolysis as well as activating growth factors.^7^ Recently, glycolysis enhancement under hypoxia have been illustrated to have close relation to invasion and metastasis in solid tumours such as NSCLC.^8^ It can be seen that clarifying molecular mechanism underlying glycolysis is of great significance for figuring out therapeutic strategies for NSCLC.

Circular RNAs (circRNAs) are a kind of special RNAs lacking 5′ and 3′ ends and forming a closed ring through covalent bond.^9^ Such special circular structure makes circRNAs resistant to exonuclease RNase R, and its half‐life in cells exceeds 48 h, exhibiting excellent stability.^10^ CircRNAs facilitates or represses tumour metastasis through modulating related target genes, affecting EMT, tumour immune escape, tumour angiogenesis, DNA methylation modification, exosome function, etc.^11^ Liu et al. have illustrated that circ_ 0001649 presents downregulation in NSCLC tumour tissue, and circ_0001649 level has relation to TNM stage, lymph node metastasis and prognosis.^12^ CircPUM1 elevates cell cycle D1 level and facilitates lung adenocarcinoma cell proliferation and glycolysis.^13^, ^14^ Circ_0007534 presents upregulation in NSCLC patients and can facilitate NSCLC metastasis through modulating EMT.^15^ CircSLC25A16 promotes glycolysis in NSCLC through epigenetic modifications.^16^ The above reports have illustrated that circRNAs exert multiple roles in NSCLC occurrence and development.

The sequencing results in literature have illustrated that multiple circRNAs exhibit upregulation in NSCLC.^17^ We attempted to figure out which circRNA exerts a key role in NSLCL progression and elucidate its molecular mechanism underlying glycolysis. Here, we found that circDHTKD1 (hsa_circ_0003074) is significantly upregulated in NSCLC. EIF4A3‐triggered circDHTKD1 upregulation facilitates NSCLC glycolysis through recruiting m6A reader IGF2BP2 to stabilize PFKL. Our research may provide a novel insight for seeking targeted therapy plans of NSCLC.

## 2 MATERIALS AND METHODS

### 2.1 Tissue specimens

We acquired 15 NSCLC tissue specimens and paired adjacent noncancerous lung tissue specimens. Pathological and histological features of NSCLC cases received confirmation based on *Revised International System for Staging Lung Cancer*. Participants had not received any chemotherapy or radiotherapy prior to specimen collection. All specimens received snap‐freezing with liquid nitrogen and storage at −80°C before RNA isolation. All cases enrolled in our research signed informed consent, and the ethics committee of our hospital granted approval for our research (AF‐SOP‐07‐1.2‐01).

### 2.2 Cell lines and culture

Human NSCLC cell lines (H1299 and A549) provided by Procell (Wuhan, China) received culture in Dulbecco's modified Eagle medium (DMEM; Hyclone, USA), 100 U/mL penicillin and 100 U/mL streptomycin at 37°C with 5% CO_2_. Control cell line BEAS‐2B provided by Procell received culture in F‐12 K medium (Sigma‐Aldrich), 100 U/mL penicillin and 100 U/mL streptomycin at 37°C with 5% CO_2_. The cells at logarithmic growth phase received collection for further use.

### 2.3 RNA isolation and RT‐qPCR

Total RNA received isolation from cells through TRIzol. MRNAs and circRNAs were reverse‐transcribed through PrimeScript RT master mix and designed primers normalizing to GAPDH. The PCR reaction was run in triplicate through 7500 Real‐Time PCR System with SYBR Premix Ex Taq II.

### 2.4 Circular characterization confirmation

Two types of primers, random and oligo (dT), were utilized for reverse transcription in same two sets of total RNA, respectively. CircRNA and mRNA levels in H1299 and A549 cells received measurement with divergent and convergent primers through RT‐qPCR. Moreover, total RNA (5 μg) received incubation with or without RNase R (5 U/μg) at 37°C for 1 h. RT‐qPCR examined circRNA and mRNA levels in H1299 and A549 cells.

### 2.5 Fluorescence in situ hybridization (FISH)

The FITC‐labelled circDHTKD1 probes were obtained from Geneseed Biotechnology (Guangzhou, China). H1299 and A549 cells received placing on coverslips, followed by fixation, permeabilization in PBS with 0.5% Triton X‐100 and dehydration in ethanol. The FISH probes received dilution (1:50), denaturation and balancing, and addition to cells at 37°C overnight. Cells received labelling with DAPI‐antifade for 10 min at room temperature post hybridization. Slides received sealing with rubber cement, placing in the dark for over 20 min and detection through fluorescence microscopy (Leica, Switzerland).

### 2.6 Cell transfection

EIF4A3 pcDNA vector (EIF4A3), circDHTKD1 pcDNA vector (circDHTKD1), PFKL pcDNA vector (PFKL) and control empty vectors (Vector) as well as short harpin RNA (shRNA) targeting EIF4A3 (sh‐EIF4A3), circDHTKD1 (sh‐circDHTKD1), IGF2BP2 (sh‐IGF2BP2) and negative control (sh‐NC) were obtained from GenePharma (Shanghai, China). H1299 and A549 cells received culture to approximately 80% confluence in plates and then received transfection with designated plasmids using Lipofectamine 3000 according to manufacturer's instructions. After 48 h, cells received collection for further use.

### 2.7 Western blotting

Total protein got extraction from cells through RIPA. Protein received isolation through SDS‐PAGE and transferring to PVDF membranes. Seal the membrane with 5% skim milk and incubate overnight with the primary antibody at 4°C. Protein bands were visualized using ECL reagent (Bio‐Rad Laboratories, Shanghai, China) after horseradish peroxidase (HRP)‐conjugated secondary antibodies (Abcam, Cambridge, UK) incubation and analysed using Quantity One software (Bio‐Rad Laboratories, Shanghai, China). The primary antibodies were as follows: anti‐EIF4A3 (ab180573, Abcam, Cambridge, UK), anti‐caspase‐3 (ab32351, Abcam, Cambridge, UK), anti‐cleaved caspase‐3 (ab2302, Abcam, Cambridge, UK), anti‐PARP1 (ab191217, Abcam, Cambridge, UK), anti‐cleaved PARP1 (ab32064, Abcam, Cambridge, UK), anti‐HK2 (ab209847, Abcam, Cambridge, UK), anti‐PFKL (ab154804, Abcam, Cambridge, UK), anti‐PFKP (ab119796, Abcam, Cambridge, UK), anti‐PFKM (ab154804, Abcam, Cambridge, UK), anti‐PKM2 (ab85555, Abcam, Cambridge, UK) and anti‐β‐actin (ab8226, Abcam, Cambridge, UK).

### 2.8 Bioinformatics

CircRNA target genes received prediction through CircInteractome (https://circinteractome.nia.nih.gov/).

### 2.9 RNA pull down

RNA pull‐down was conducted through a Pierce Magnetic Kit. EIF4A3 or IGF2BP2 received capture via streptavidin magnetic beads and received incubation with H1299 and A549 cell lysates at 4°C for 6 h. Mixture received washing and elution. The eluate received measurement through western blotting.

### 2.10 RIP

H1299 and A549 cell lysates received extraction on ice for 2 h, centrifugation and incubation with antibody‐conjugated beads for 4°C overnight. Antibody‐bead complexes received washing five or six times with cold lysis buffer. RT‐qPCR examined circDHTKD1 downstream intron mRNA enrichment in anti‐EIF4A3‐ or anti‐IgG‐immunoprecipitated complexes as well as circDHTKD1 or PEKL enrichment in anti‐IGF2BP2‐ or anti‐IgG‐immunoprecipitated complexes.

### 2.11 Colony formation

Cell proliferation was evaluated through colony formation. H1299 and A549 cells (2 × 10^3^ cells/well) received seeding into 6‐well plates for forming colonies. After 2 weeks, colonies received fixation through 4% paraformaldehyde and staining through 0.1% crystal violet for 15 min at room temperature. The colonies (>50 cells) received photographing and quantification through microscopy.

### 2.12 EdU staining

EdU cell proliferation assay kit (Ribobio) was applied for evaluating cell proliferation. The 100 μL of EdU (50 μM) received addition to per well. H1299 and A549 cells received culture for 2 h. Cell nuclei received staining with Hoechst (1 μg/mL) for 30 min. The cell proportion incorporated EdU received confirmation through fluorescence microscopy.

### 2.13 Animal models

A total of 10 BALB/c nude mice (4 weeks old, 15–20 g) weight were provided by SLARC (Shanghai, China). All animal procedures acquired permission from the Animal Care and Use Committee of The First Hospital of China Medical University. The viable A549 cells stably expressing sh‐circDHTKD1 or sh‐NC (5 × 105) received injection into nude mice right flank. The mice received sacrificing when tumours were apparent on the thirth day. The tumours received photographing and weighing followed by storage for IHC analysis. The study was conducted according to the protocol approved by the Animal Welfare Ethics Committee of China Medical University (KT20240739).

### 2.14 Immunochemistry (IHC)

Mouse tumour tissue was sectioned at 5 μm, deparaffinized and rehydrated. For antigen retrieval, sections are processed in a laboratory microwave oven in citrate buffer (pH 6.0) at 95°C for 20 min followed by washing in PBS. For immunohistochemistry, sections were incubated with anti‐Ki67 (ab15580, Abcam, USA) primary antibody overnight at 4°C after quenching endogenous peroxidase activity and blocking with normal goat serum, followed by incubation with HRP‐conjugated secondary antibody. Immunostaining was observed with DAB (Dako, Denmark). Sections are then counterstained with haematoxylin and then examined using a light microscope.

### 2.15 Measurement of glucose consumption, lactate production and ATP level

For glucose uptake assays, glucose consumption is performed using a colorimetric glucose assay kit (EIAGLUC, ThermoFisher, USA) and normalized according to cell number. For the lactate assay, we use the lactate assay kit (ab65330, Abcam, Cambridge, UK) to detect the lactate concentration in whole cell lysis according to the manufacturer's instructions. For ATP testing, we use the ATP detection kit (ab113849, Abcam, Cambridge, UK) to measure the level of ATP.

### 2.16 Measurement of extracellular acidification rate (ECAR)

A Seahorse XF96 flux analyser (Seahorse Bioscience) was utilized for assessing glycolysis through monitoring ECAR under manufacturer's guidance. In brief, H1299 and A549 cells received seeding into 96‐well plates at 1 × 10^4^ cells per well overnight. The cells received incubation with unbuffered medium followed by injection with sequential inhibitors: 10 mM of glucose, 1 mM of oligomycin and 80 mM of 2‐deoxyglucose (2‐DG).

### 2.17 Measurement of RNA stability

For blocking transcription, 2 mg/mL actinomycin D or negative control DMSO was added into H1299 cell culture medium. At 3 and 6 h after H1299 cells receiving treatment with actinomycin D, RT‐qPCR determined RNA levels in H1299 cells.

### 2.18 Statistical analysis

Statistical analysis was conducted using SPSS 22.0. Comparison between groups was performed using *t*‐test. Comparison of three or more groups was carried out via one‐way analysis of variance. Pearson correlation assessed association of circDHTKD1 with EIF4A3 or PFKL level in NSCLC tissue specimens. All assays were carried out in triplicate. The difference was statistically significant once *p* < 0.05.

## 3 RESULTS

### 3.1 CircDHTKD1 presents expression imbalance in NSCLC

**FIGURE 1:**
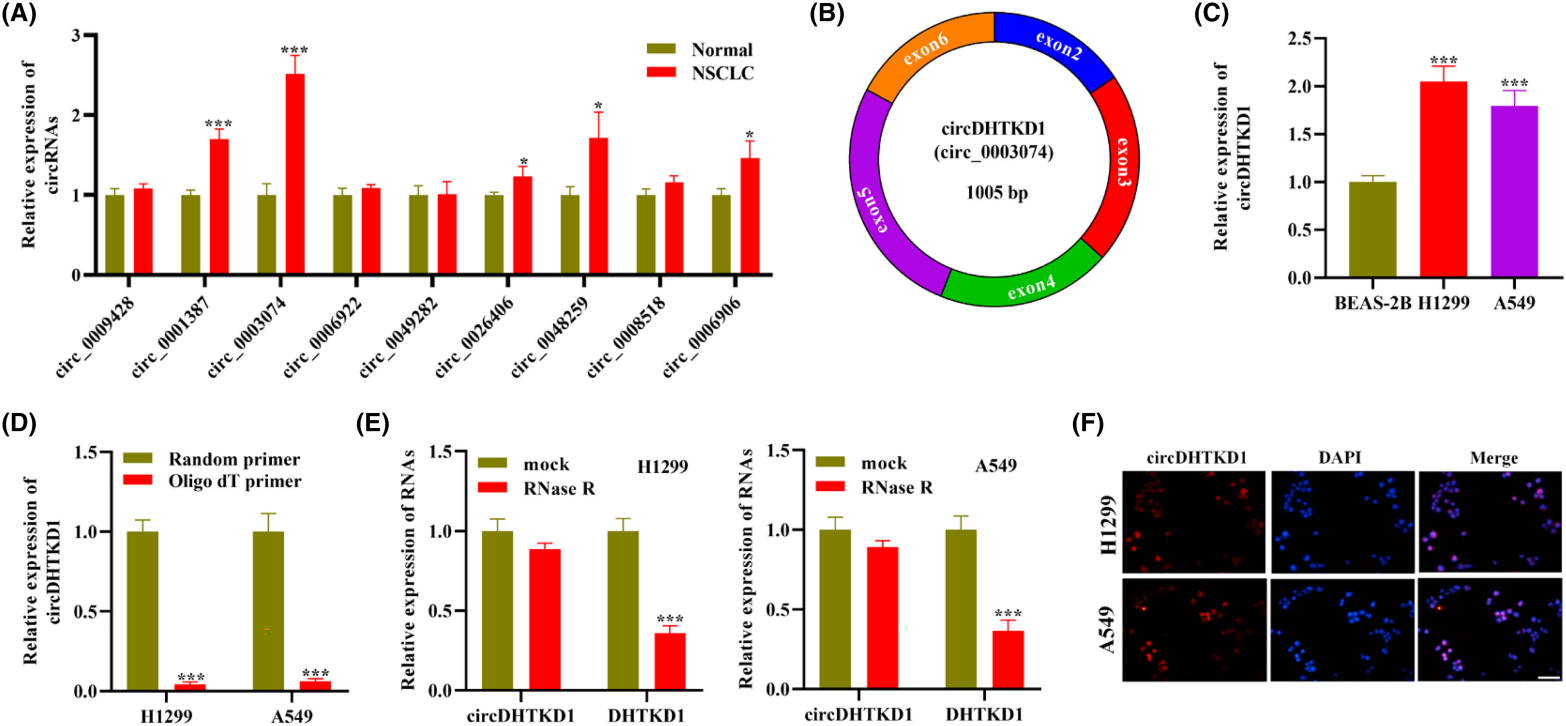
Observation for circDHTKD1 expression and characteristics in NSCLC cells. (A) RT‐qPCR examined circRNA levels in NSCLC tissue specimens and normal controls. (B) Looping diagram of circDHTKD1. (C) RT‐qPCR examined circDHTKD1 level in NSCLC cell lines (H1299 and A549) and control cell line BEAS‐2B. (D) RT‐qPCR examined circDHTKD1 level after amplification with Oligo dT or random primers. (E) RNase R digestion assay detected circular and linear DHTKD1 degradation degree in NSCLC cells. (F) The localization of circDHTKD1 in NSCLC cells through FISH. Scale bars, 1000 μm. *p < 0.05, ***p < 0.001.

The sequencing results have illustrated that several circRNAs exhibit upregulation in NSCLC.^17^ To figure out which circRNA exerts a key role in NSLCL progression, we measured levels of these circRNAs in NSCLC tissue specimens through RT‐qPCR. As a result, circ_0003074 exhibited the most upregulation in NSLCL tissue (Figure 1A). Thus we chose circ_0003074 as key molecule in our further assays. Additionally, loop diagram of circ_0003074 illustrated that circ_0003074 received formation through reverse splicing of DHTKD1 exon 2–6, with a total length of 1005 bp, termed as circDHTKD1 (Figure 1B). RT‐qPCR depicted that circDHTKD1 exhibited upregulation in NSCLC cell lines (H1299 and A549) relative to control cell line BEAS‐2B (Figure 1C). RT‐qPCR depicted that circDHTKD1 presented depletion after amplified with Oligo dT primers relative to random primers (Figure 1D).

Circular structure makes circRNAs resistant to exonuclease RNase R,^10^ thus we conducted RNase R digestion assay in NSCLC cells. Linear RNA transcript (DHTKD1 mRNA) exhibited a marked degradation relative to circDHTKD1 in H1299 and A549 cells (Figure 1E). These results further confirmed circular characterization and stability of circDHTKD1. Furthermore, we assessed circDHTKD1 localization in NSCLC cells through FISH. As a result, circDHTKD1 exhibited predominately cytoplasmic distribution in H1299 and A549 cells (Figure 1F). Collectively, circDHTKD1 exhibits upregulation and stable circular nature in NSCLC cells.

### 3.2 EIF4A3 enhances circDHTKD1 expression

**FIGURE 2:**
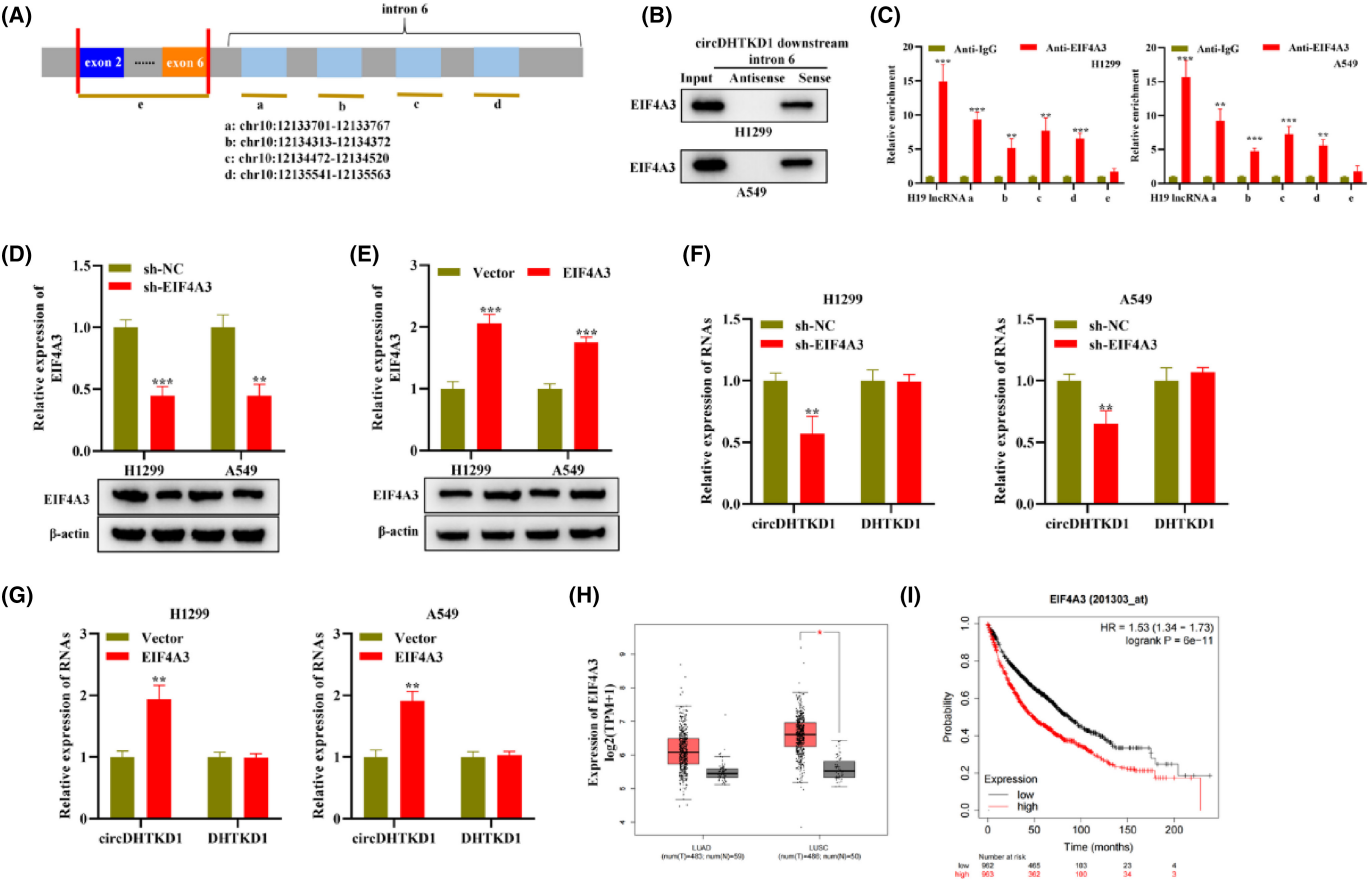
EIF4A3 enhanced circDHTKD1 expression. (A) Binding sequence of EIF4A3 on DHTKD1 pre‐mRNA. (B) RNA pull‐down followed by western blotting detected EIF4A3 enrichment in complexes bound to circDHTKD1 downstream intron mRNA sense or circDHTKD1 downstream intron mRNA antisense in NSCLC cells. (C) RIP followed by RT‐qPCR detected circDHTKD1 flanking region enrichment in complexes bound to anti‐EIF4A3 or anti‐IgG in H1299 cells. (D) RT‐qPCR and western blotting detected knockdown efficacy of sh‐NC or sh‐EIF4A3 in NSCLC cells. (E) RT‐qPCR and western blotting detected overexpression efficacy of vector or EIF4A3 in NSCLC cells. (F, G) RT‐qPCR detected circular or linear DHTKD1 level in NSCLC cells under indicated transfection. (H) Pearson correlation assessed association of circDHTKD1 and EIF4A3 levels in NSCLC tissue specimens. (I) Kmplot assessed association of EIF4A3 with survival of NSCLC patients. *p < 0.05, **p < 0.01, ***p < 0.001.

We attempted to clarify whether RBP participates in upregulating circDHTKD1. According to CircInteractome, EIF4A3, DGCR and U2AF65 may bind to flanking region of circDHTKD1. Among them, EIF4A3 possess the most binding sequences, and EIF4A3, a vital component of exon junction complex, exerts a role in splicing pre‐mRNA. Thus, we further attempted to elucidate specific impact of EIF4A3 on circDHTKD1. We obtained binding sequence of EIF4A3 on DHTKD1 pre‐mRNA (Figure 2A). RNA pull down depicted that EIF4A3 remarkably enriched in pulled‐down products by circDHTKD1 downstream intron mRNA sense rather than circDHTKD1 downstream intron mRNA antisense in H1299 and A549 cells (Figure 2B), supporting a binding of EIF4A3 to circDHTKD1 downstream intron mRNA in NSCLC cells. RIP illustrated that fragments a, b, c and d exhibited enrichment in complexes immunoprecipitated with anti‐EIF4A3 rather than anti‐IgG in H1299 cells (Figure 2C), suggesting that EIF4A3 can bind to flanking region of circDHTKD1 through binding fragment obtained from bioinformatics.

Moreover, H1299 and A549 cells received transfection using sh‐NC or sh‐EIF4A3. RT‐qPCR and western blotting confirmed the successful knockdown of EIF4A3 in sh‐EIF4A3‐transfected NSCLC cells (Figure 2D). H1299 and A549 cells received transfection using empty vector or pcDNA‐EIF4A3. RT‐qPCR and western blotting confirmed the successful upregulation of EIF4A3 in EIF4A3‐transfected NSCLC cells (Figure 2E). Furthermore, RT‐qPCR depicted that circDHTKD1 exhibited upregulation in H1299 and A549 cells under EIF4A3 knockdown whereas exhibited downregulation under EIF4A3 overexpression, meanwhile, DHTKD1 showed no level change in NSCLC cells (Figure 2F,G), suggesting that EIF4A3 exerts a positive regulation on circDHTKD1 rather than DHTKD1. Pearson correlation illustrated that circDHTKD1 level possessed a positive relation to EIF4A3 level in NSCLC tissue specimens (Figure 2H). Additionally, Kmplot illustrated that high‐level EIF4A3 had a close relation to shorter survival of NSCLC patients (Figure 2I). Collectively, EIF4A3 upregulates circDHTKD1 in NSCLC cells.

### 3.3 CircDHTKD1 facilitates NSCLC malignancy

**FIGURE 3:**
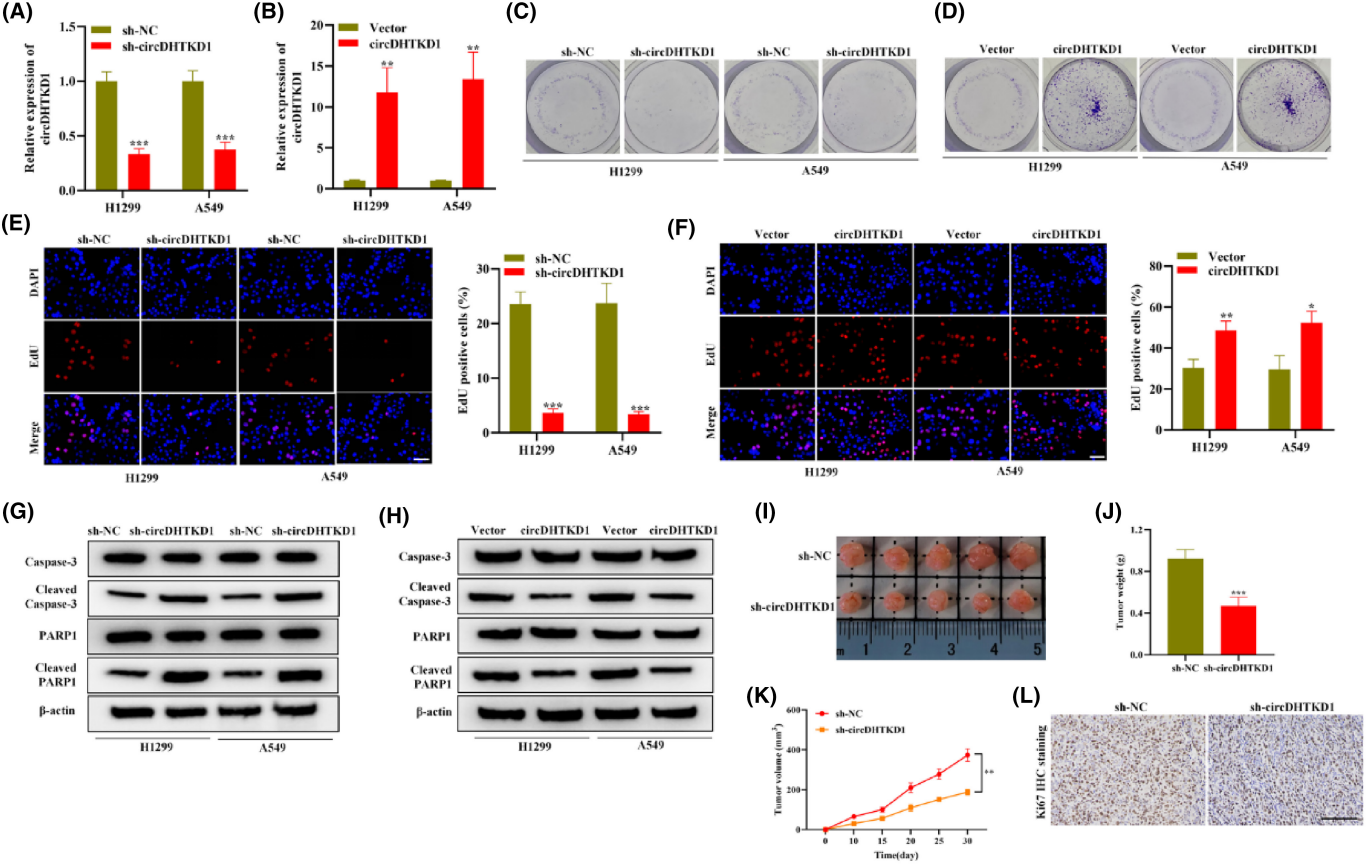
CircDHTKD1 facilitated NSCLC malignancy. (A) RT‐qPCR detected knockdown efficacy of sh‐NC or sh‐circDHTKD1 in NSCLC cells. (B) RT‐qPCR detected overexpression efficacy of vector or circDHTKD1 in NSCLC cells. (C, D) Colony formation assessed NSCLC cell proliferation through transfection of sh‐NC or sh‐circDHTKD1 as well as vector or circDHTKD1. (E, F) EdU staining assessed NSCLC cell proliferation under indicated transfection. Scale bars, 1000 μm. (G, H) Western blotting assessed NSCLC cell apoptosis‐related protein levels under indicated transfection. (I) Representative tumour images of mice in sh‐NC or sh‐circDHTKD1 group. (J) Tumour weight of mice in each group. (K) Tumour volume of mice in each group. (L) IHC detected Ki67 level in mouse lung tissue in each group. Scale bars, 100 μm. *p < 0.05, **p < 0.01, ***p < 0.001.

Due to circDHTKD1 upregulation in NSCLC, we hypothesized that circDHTKD1 may function as an oncogene in NSCLC cellular phenotypes. Thus, we carried out loss‐ and gain‐of‐function assays. First, H1299 and A549 cells received transfection using sh‐NC or sh‐circDHTKD1 as well as empty vector or pcDNA‐circDHTKD1. RT‐qPCR confirmed the successful knockdown or upregulation of circDHTKD1 in sh‐circDHTKD1 or circDHTKD1‐transfected NSCLC cells (Figure 3A,B). Then, colony formation and EdU staining assessed NSCLC cell proliferative ability. As a result, circDHTKD1 silencing resulted in a remarkable colony amount reduction whereas circDHTKD1 elevation resulted in a remarkable colony amount increase in H1299 and A549 cells (Figure 3C,D). CircDHTKD1 silencing resulted in a remarkable EdU‐positive cell proportion reduction whereas circDHTKD1 elevation resulted in a remarkable EdU‐positive cell proportion increase in H1299 and A549 cells (Figure 3E,F).

Furthermore, western blotting assessed apoptosis‐related protein abundances in NSCLC cells. As a result, circDHTKD1 silencing led to increased cleaved‐caspase‐3/caspase‐3 and cleaved‐PARP1/PARP1 ratios whereas circDHTKD1 elevation led to decreased cleaved‐caspase‐3/caspase‐3 and cleaved‐PARP1/PARP1 ratios in H1299 and A549 cells (Figure 3G,H). The above results suggest that circDHTKD1 facilitates NSCLC cell proliferative ability and suppresses NSCLC cell apoptosis. Subsequently, we attempted to clarify whether circDHTKD1 acted as oncogene in vivo. Mice received injection of A549 cells stably transfected with sh‐circDHTKD1 or sh‐NC. We discovered that circDHTKD1 silencing led to tumour size and weight reduction in NSCLC mice (Figure 3I,J). Moreover, IHC assessed proliferation‐related protein Ki67 level in NSCLC mice. As a result, circDHTKD1 silencing caused Ki67 downregulation in NSCLC mice (Figure 3K). Collectively, circDHTKD1 exerts a promoting influence on NSCLC cell malignant phenotypes and tumour growth.

### 3.4 CircDHTKD1 regulates glucose metabolism in NSCLC

**FIGURE 4:**
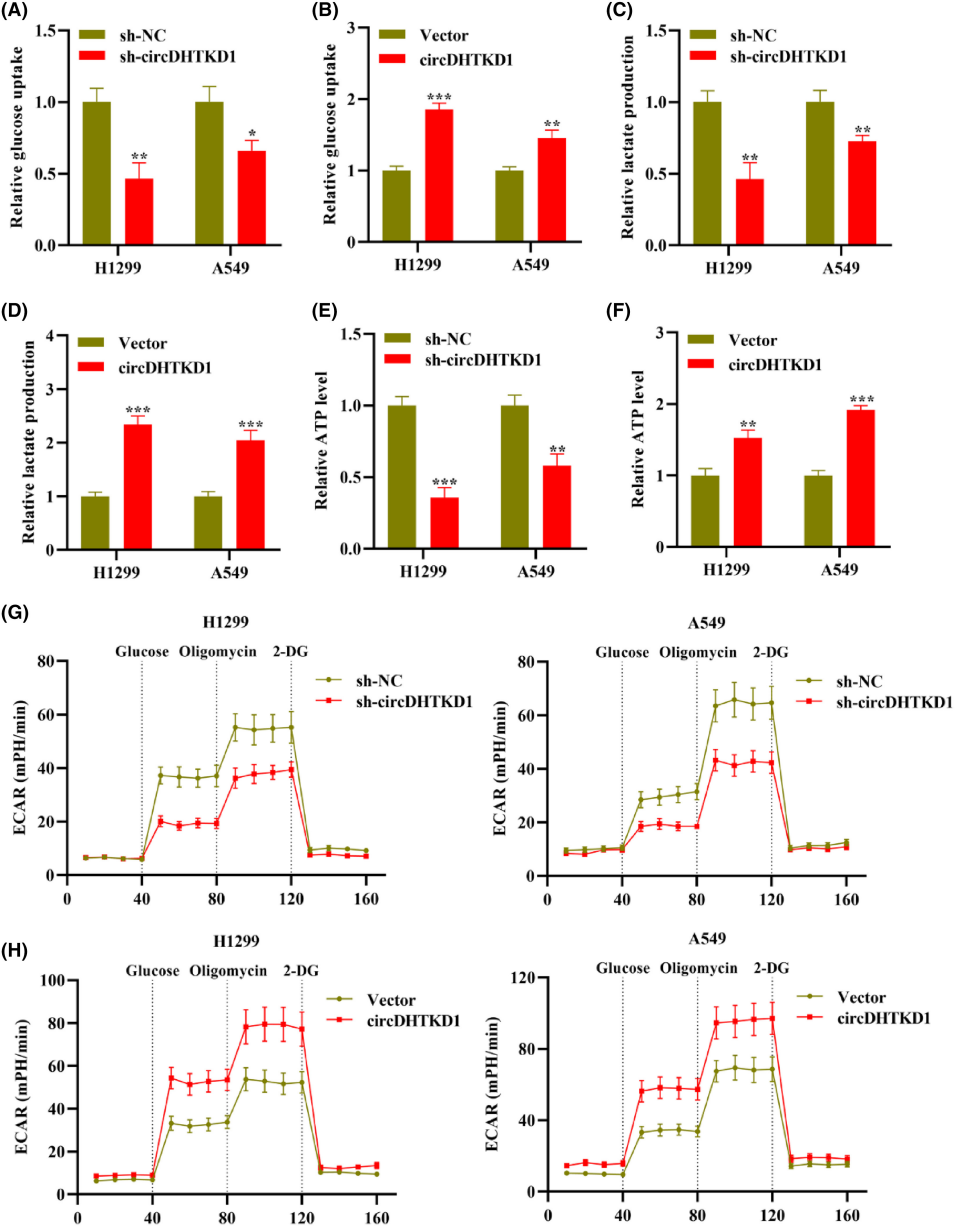
CircDHTKD1 regulated glucose metabolism in NSCLC. (A, B) The glucose kit measured NSCLC glucose uptake through transfection of sh‐NC or sh‐circDHTKD1 as well as vector or circDHTKD1. (C, D) The lactate kit measured NSCLC lactate production under indicated transfection. (E, F) The ATP kit measured NSCLC ATP level under indicated transfection. (G, H) NSCLC ECAR changes under indicated transfection. *p < 0.05, **p < 0.01, ***p < 0.001.

Tumour cells possess characterization with upregulated metabolism in glycolysis and lactate production, even in presence of massive oxygen, termed as aerobic glycolysis (Warburg effect).^5^ Thus, we attempted to clarify circDHTKD1 impact on glucose metabolism in NSCLC. Glucose kits illustrated that circDHTKD1 silencing led to glucose uptake downregulation whereas circDHTKD1 elevation led to glucose uptake upregulation in H1299 and A549 cells (Figure 4A,B). Additionally, lactate kits illustrated that circDHTKD1 silencing led to lactate production downregulation whereas circDHTKD1 elevation led to lactate production upregulation in H1299 and A549 cells (Figure 4C,D). Furthermore, ATP kits illustrated that circDHTKD1 silencing led to ATP downregulation whereas circDHTKD1 elevation led to ATP upregulation in H1299 and A549 cells (Figure 4E,F). Moreover, ECAR kits illustrated that circDHTKD1 silencing led to ECAR downregulation whereas circDHTKD1 elevation led to ECAR upregulation in H1299 and A549 cells (Figure 4G,H). Collectively, circDHTKD1 exerts a promoting influence on NSCLC glucose metabolism.

### 3.5 CircDHTKD1 facilitates NSCLC glycolysis through PFKL

**FIGURE 5:**
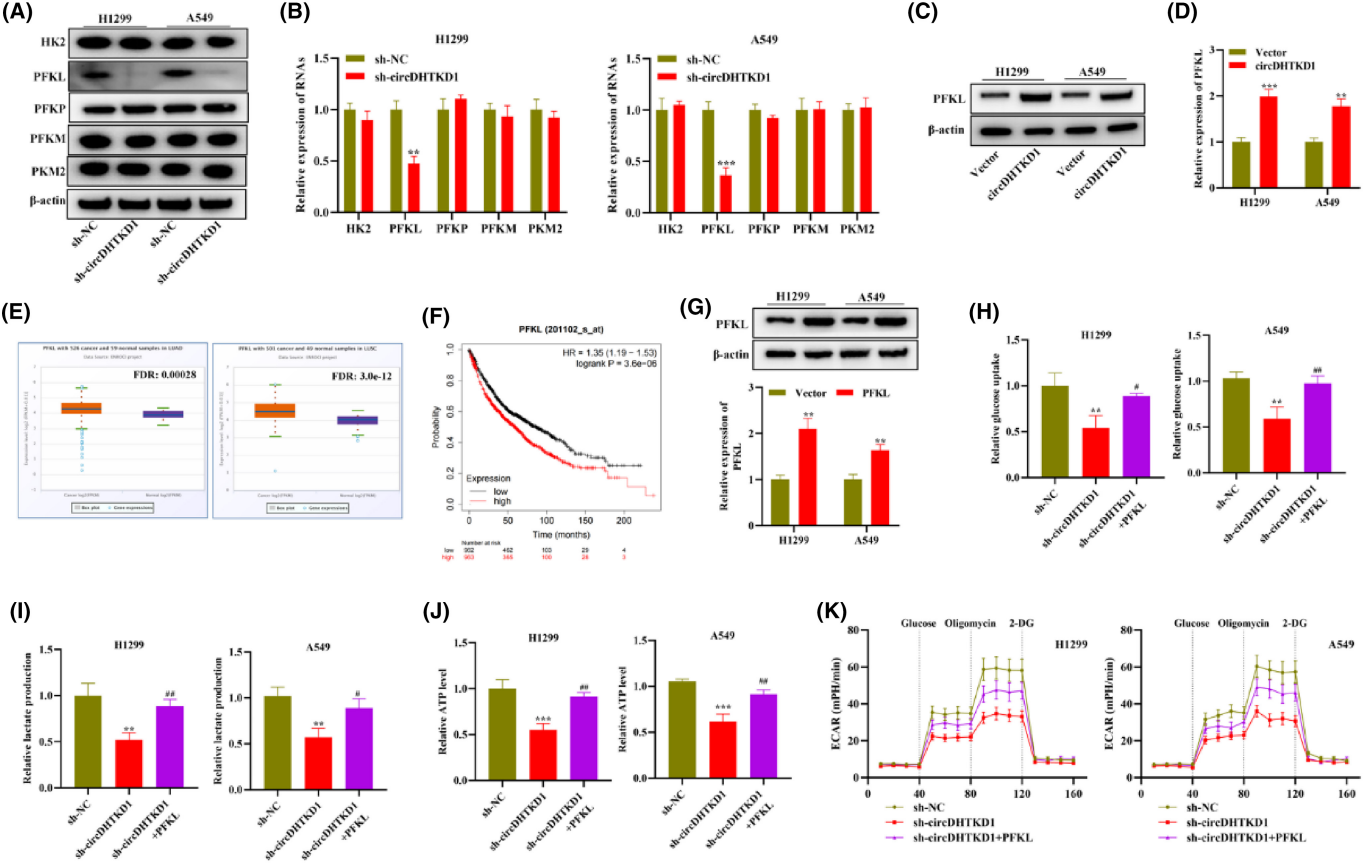
CircDHTKD1 facilitated NSCLC glycolysis through PFKL. (A, B) Western blotting and RT‐qPCR examined level changes of three rate‐limiting enzymes in sh‐NC‐ or sh‐circDHTKD1‐transfected NSCLC cells. (C, D) Western blotting and RT‐qPCR examined PFKL level in vector‐ or circDHTKD1‐transfected NSCLC cells. (E) Pearson correlation assessed association of circDHTKD1 and PFKL levels in NSCLC tissue specimens. (F) Kmplot assessed association of PFKL with survival of NSCLC patients. (G) RT‐qPCR and western blotting detected overexpression efficacy of vector or PFKL in NSCLC cells. (H) The glucose kit measured NSCLC glucose uptake through transfection of sh‐NC, sh‐circDHTKD1 or sh‐circDHTKD1+PFKL. (I) The lactate kit measured NSCLC lactate production under indicated transfection. (J) The ATP kit measured NSCLC ATP level under indicated transfection. (K) NSCLC ECAR changes under indicated transfection. **p < 0.01, ***p < 0.001, compared with the sh‐NC group; #*p* < 0.05, ##*p* < 0.01, compared with the sh‐circDHTKD1 group.

We attempted to clarify mechanism of circDHTKD1 modulating glucose metabolism, thus we measured level changes of three rate‐limiting enzymes (HK2, PFK1 and PKM2) during glycolysis, among which PFK1 included PFKL, PFKP and PFKM. Western blotting and RT‐qPCR illustrated that circDHTKD1 knockdown attenuated PFKL protein and mRNA levels rather than other enzymes in H1299 and A549 cells (Figure 5A,B). On the contrary, circDHTKD1 overexpression elevated PFKL protein and mRNA levels in H1299 and A549 cells (Figure 5C,D). Pearson correlation illustrated that circDHTKD1 level possessed a positive relation to PFKL level in NSCLC tissue specimens (Figure 5E). Additionally, Kmplot illustrated that high‐level PFKL had a close relation to shorter survival of NSCLC patients (Figure 5F). H1299 and A549 cells received transfection using empty vector or pcDNA‐PFKL. RT‐qPCR and western blotting confirmed the successful upregulation of PFKL in PFKL‐transfected NSCLC cells (Figure 5G). Subsequently, we carried out rescue assays on glucose metabolism. As a result, reduction in glucose uptake, lactate production, ATP level and ECAR in H1299 and A549 cells due to circDHTKD1 silencing was reversed through PFKL upregulation (Figure 5H–K). Collectively, circDHTKD1 exerts a promoting influence on NSCLC glucose metabolism through PFKL upregulation.

### 3.6 CircDHTKD1 recruits IGF2BP2 to stabilize PFKL

**FIGURE 6:**
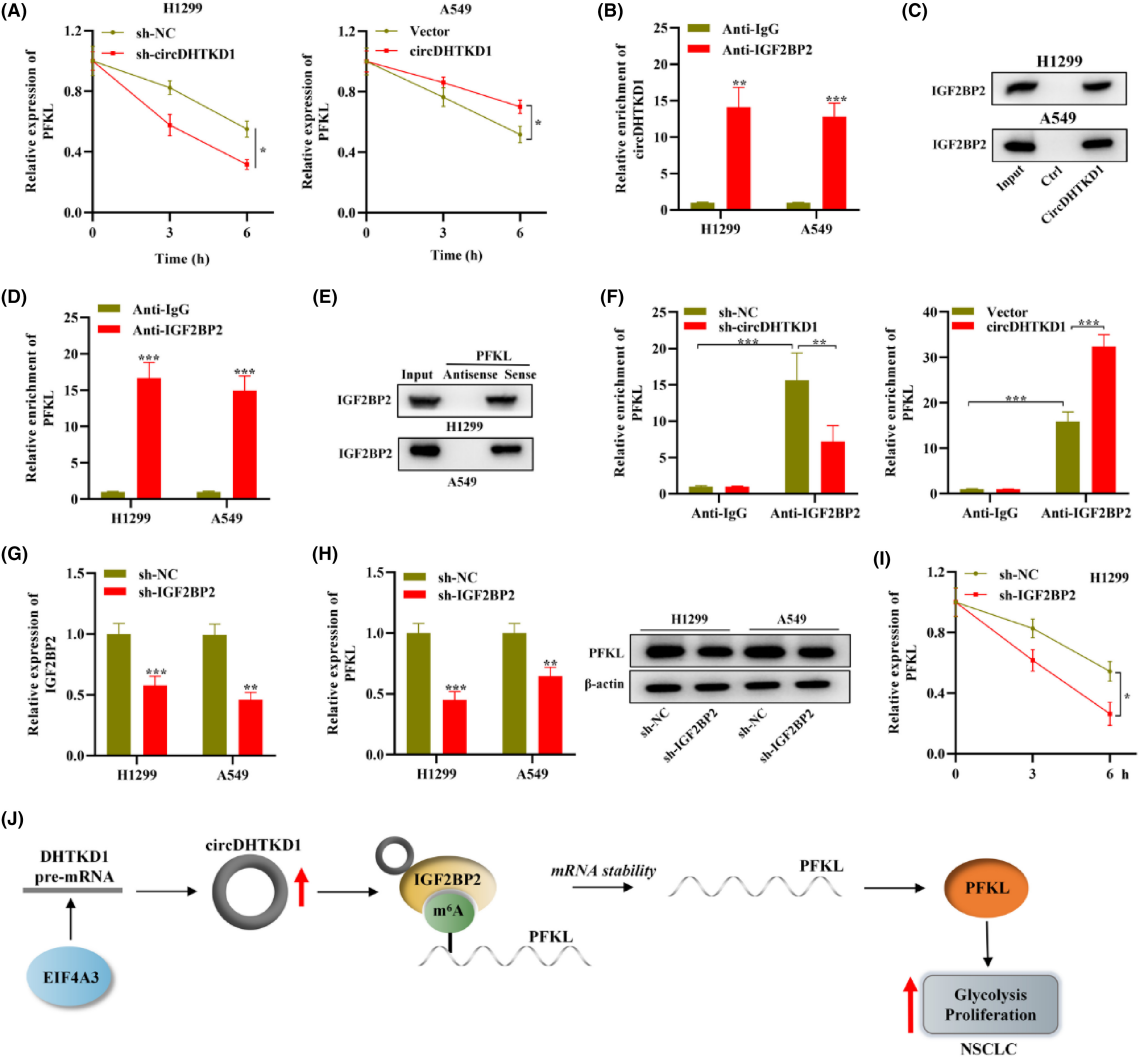
CircDHTKD1 recruited IGF2BP2 to stabilize PFKL. (A) RT‐qPCR detected PFKL mRNA half‐life in sh‐NC‐ or sh‐circDHTKD1‐transfected NSCLC cells. (B) RIP followed by RT‐qPCR detected circDHTKD1 enrichment in complexes bound to anti‐IGF2BP2 or anti‐IgG in NSCLC cells. (C) RNA pull‐down followed by western blotting detected IGF2BP2 enrichment in complexes bound to circDHTKD1 or controls in NSCLC cells. (D) RIP followed by RT‐qPCR detected PFKL enrichment in complexes bound to anti‐IGF2BP2 or anti‐IgG in NSCLC cells. (E) RNA pull‐down followed by western blotting detected IGF2BP2 enrichment in complexes bound to PFKL sense or PFKL antisense in NSCLC cells. (F) RIP followed by RT‐qPCR detected PFKL enrichment in complexes bound to anti‐IGF2BP2 or anti‐IgG in NSCLC cells under indicated transfection. (G) RT‐qPCR detected knockdown efficacy of sh‐NC or sh‐IGF2BP2 in NSCLC cells. (H) RT‐qPCR and western blotting measured PFKL level in sh‐NC‐ or sh‐IGF2BP2‐transfected NSCLC cells. (I) RT‐qPCR detected PFKL mRNA half‐life in sh‐NC‐ or sh‐IGF2BP2‐transfected NSCLC cells.

We attempted to elucidate molecular mechanism of circDHTKD1 regulating PFKL. RT‐qPCR examined PFKL mRNA half‐life in H1299 cells. As a result, circDHTKD1 knockdown facilitated PFKL mRNA degradation whereas circDHTKD1 overexpression suppressed mRNA degradation in H1299 cells (Figure 6A), suggesting that circDHTKD1 stabilizes PFKL in NSCLC cells. According to previous literature, m6A “reader” can regulate PFKL stability after binding to PFKL,^18^ and circAtlas website depicts that circDHTKD1 can bind to m6A reading protein IGF2BP2, thus we hypothesized that circDHTKD1 may stabilize PFKL through IGF2BP2. Subsequently, we carried out mechanism assays to validate such hypothesis. First, RIP depicted that circDHTKD1 exhibited a marked enrichment in complexes co‐immunoprecipitated through anti‐IGF2BP2 rather than anti‐IgG in H1299 and A549 cells (Figure 6B). RNA pull‐down illustrated that IGF2BP2 protein exhibited enrichment in complexes pulled‐down by circDHTKD1 rather than control in H1299 and A549 cells (Figure 6C), supporting the binding of circDHTKD1 to IGF2BP2.

Then, RIP depicted that PFKL exhibited a marked enrichment in complexes co‐immunoprecipitated through anti‐IGF2BP2 rather than anti‐IgG in H1299 and A549 cells (Figure 6D). RNA pull‐down illustrated that IGF2BP2 protein exhibited enrichment in complexes pulled‐down by PFKL sense rather than PFKL antisense in H1299 and A549 cells (Figure 6E), supporting the binding of PFKL to IGF2BP2. Moreover, RIP depicted that PFKL enrichment in complexes co‐immunoprecipitated through anti‐IGF2BP2 in H1299 cells exhibited a marked reduction under circDHTKD1 knockdown whereas exhibited a marked elevation under circDHTKD1 overexpression (Figure 6F), suggesting that circDHTKD1 facilitates the binding of PFKL to IGF2BP2. H1299 and A549 cells received transfection using sh‐NC or sh‐IGF2BP2. RT‐qPCR confirmed the successful knockdown of IGF2BP2 in sh‐IGF2BP2‐transfected NSCLC cells (Figure 6G). RT‐qPCR and western blotting depicted that PFKL exhibited downregulation in H1299 and A549 cells under IGF2BP2 knockdown (Figure 6H). Furthermore, RT‐qPCR depicted that IGF2BP2 knockdown facilitated PFKL mRNA degradation in H1299 cells (Figure 6I), suggesting that IGF2BP2 stabilizes PFKL in NSCLC cells. Collectively, circDHTKD1 stabilizes PFKL through recruiting m6A reader IGF2BP2 in NSCLC.

## 4 DISCUSSION

Multiple circRNAs presents stable and abnormal expression in NSCLC, with good sensitivity and specificity, and has been illustrated to have relation to unfavourable prognosis,^19^, ^20^, ^21^ providing a new choice for early diagnosis and development of prognostic markers for NSCLC. CircDHTKD1 have been revealed as an oncogene to facilitate metastasis in bladder cancer as well as oral squamous cell carcinoma.^22^, ^23^ Herein, circDHTKD1 exhibited upregulation in NSLCL tissue and cells. Moreover, we confirmed circular characterization and stability of circDHTKD1. Moreover, circDHTKD1 knockdown repressed NSCLC cell proliferative ability as well as tumour growth. These findings suggested that circDHTKD1 may serve as an oncogene in NSCLC.

RNA binding proteins (RBPs) are a class of proteins that can specifically bind to RNAs in cells, and then get involvement in a series of post‐transcriptional regulation functions such as RNA splicing, transport, translation and localization.^24^ Thus RBP can get involvement in formation, post‐transcriptional regulation and translation of circRNAs through interaction, thus affecting circRNA function.^25^ We suspected that circDHTKD1 exhibited upregulation in such manner. EIF4A3, a member of EIF4A eukaryotic translation initiation factor family and a vital component of exon junction complex, exerts a role in splicing pre‐mRNA.^26^ EIF4A3 exerts a vital role in tumorigenesis, development, oncogene expression, tumour cell invasion and metastasis.^27^ It has been revealed that EIF4A3 exhibits upregulation in a variety of tumours and can facilitate malignant progression,^28^, ^29^ indicating that EIF4A3 can exert a carcinogenic role in cancers. Herein, through bioinformatics, we discovered that EIF4A3 may bind to flanking region of circDHTKD1. Mechanism assays illustrated that EIF4A3 bound to flanking region of circDHTKD1 through binding fragment. Additionally, EIF4A3 exerted a positive modulation on circDHTKD1 level in NSCLC cells; circDHTKD1 level possessed a positive relation to EIF4A3 level in NSCLC tissue specimens. Furthermore, high‐level EIF4A3 had a close relation to shorter survival of NSCLC patients. These findings suggested that EIF4A3 may exert as a carcinogenic RBP to upregulate circDHTKD1 in NSCLC.

Aerobic glycolysis is one of the 10 characteristics of tumours.^30^ Normal cells take oxidative phosphorylation as major energy supply mode under sufficient oxygen, and glycolysis as major energy supply mode under hypoxia; nevertheless, tumour cells still rely on glycolysis, an inefficient way of production, even under sufficient oxygen.^31^ Recently, glycolysis enhancement under hypoxia have been illustrated to have close relation to invasion and metastasis in solid tumours such as NSCLC.^8^ Herein, circDHTKD1 silencing led to reduction in glucose uptake, lactate production, ATP level and ECAR in NSCLC cells. These findings suggested that circDHTKD1 exerts a promoting influence on NSCLC glucose metabolism.

Phosphofructokinase 1 (PFK1), a rate‐limiting enzyme in the second step of glycolysis pathway, can catalyse the phosphorylation of fructose‐6‐phosphate (F‐6‐P) to fructose‐l, 6‐diphosphate (F‐1, 6‐P).^32^ PFK1 includes three subtypes (PFKM, PFKL and PFKP), which are named for their dominant expression in muscle, liver and platelets.^33^ Additionally, PFK1 exhibits upregulation in LC tumour tissue,^34^ and can modulate metabolism and facilitate LC cell proliferation through RAS signalling pathway,^35^ whereas which subtype exerts a key role in NSCLC remains elusive. Herein, circDHTKD1 exerted a positive modulation on PFKL level rather than other enzymes in NSCLC cells; circDHTKD1 level possessed a positive relation to PFKL level in NSCLC tissue specimens; high‐level PFKL had a close relation to shorter survival of NSCLC patients. Furthermore, rescue assays revealed that PFKL upregulation countervailed inhibitory impact on circDHTKD1 silencing glucose uptake, lactate production, ATP level and ECAR in NSCLC cells. These findings suggested that circDHTKD1 exerts a promoting influence on NSCLC glucose metabolism through PFKL upregulation.

IGF2BP2 is an RBP that can recognize m6A modification, and IGF2BP2 has relation to unfavourable prognosis of multiple tumours.^36^, ^37^ According to previous literature, m6A “reader” can regulate PFKL stability after binding to PFKL,^18^ and circAtlas website depicts that circDHTKD1 can bind to m6A reading protein IGF2BP2. Herein, mechanism assays illustrated that circDHTKD1 stabilized PFKL and bound to IGF2BP2 in NSCLC cells. Meanwhile, PFKL bound to IGF2BP2 and stabilized PFKL in NSCLC cells. Additionally, circDHTKD1 facilitated the binding of PFKL to IGF2BP2. These findings suggested that circDHTKD1 stabilizes PFKL through recruiting m6A reader IGF2BP2 in NSCLC.

In this study, we found that the EIF4A3‐circDHTKD1‐IGF2BP2‐PFKL axis plays an important regulatory role in the proliferation and glycolysis of NSCLC. Studies have shown that EIF4A3‐IGF2BP2 is an important pathway in prostate cancer,^38^ suggesting that the EIF4A3‐circDHTKD1‐IGF2BP2‐PFKL axis may have a potential function in other cancers, which warrants further investigation. The mechanism of action of circDHTKD1 in NSCLC cells was thoroughly examined in this study, and it was demonstrated that by creating xenografts, circDHTKD1 promotes tumour growth in vivo in a mouse model. Nevertheless, further research is necessary to fully prove the mechanism EIF4A3‐circDHTKD1‐IGF2BP2‐PFKL axis at the in vivo level, which has not yet been covered. In addition, we used only two NSCLC cell lines to demonstrate the mechanism of action of circDHTKD1, and the lack of data from clinical samples of primary tumours in this study. In future studies, we will further explore circDHTKD1 in other cell lines and clinical samples, including the sponge effect of circDHTKD1 on miRNA, as well as related downstream target genes related to other biological events. The signaling pathway of our studies is shown in Figure 6J.

In conclusion, circDHTKD1 exhibits upregulation in NSCLC. We innovatively validate that EIF4A3‐triggered circDHTKD1 upregulation facilitates NSCLC glycolysis through recruiting m6A reader IGF2BP2 to stabilize PFKL, providing a new direction for seeking targeted therapy plans of NSCLC.

## AUTHOR CONTRIBUTIONS

**Zhenghua Liu:** Data curation (lead); formal analysis (lead); investigation (lead); methodology (lead); resources (lead); writing – original draft (lead). **Wenya Li:** Formal analysis (equal); methodology (equal); resources (equal); software (equal). **Ziyi Wang:** Data curation (supporting); formal analysis (supporting); resources (supporting). **Qiwei Yang:** Data curation (supporting); formal analysis (equal); methodology (supporting); software (equal). **Liang Chen:** Data curation (equal); formal analysis (equal); software (equal). **Weiyang Chen:** Data curation (equal); formal analysis (equal); methodology (equal). **Xiaohan Qu:** Conceptualization (lead); funding acquisition (lead); project administration (lead); writing – review and editing (lead).

## FUNDING INFORMATION

This work was supported by the Natural Science Foundation of Liaoning Province (No. 2021‐MS‐168).

## CONFLICT OF INTEREST STATEMENT

The authors have no conflict of interest.

## CONSENT FOR PUBLICATION

All authors are aware of the content of this paper and agree to publish.

## Data Availability

The data generated during the current study are available from the corresponding author on request.

## References

[1] Rammal S , Kourie HR , Jalkh N , et al. Molecular pathogenesis of hereditary lung cancer: a literature review. Pharmacogenomics. 2021;22(12):791‐803. 34410147 10.2217/pgs-2020-0150

[2] Relli V , Trerotola M , Guerra E , Alberti S . Abandoning the notion of non‐small cell lung cancer. Trends Mol Med. 2019;25(7):585‐594. 31155338 10.1016/j.molmed.2019.04.012

[3] Goldstraw P , Chansky K , Crowley J , et al. The IASLC lung cancer staging project: proposals for revision of the TNM stage groupings in the forthcoming (eighth) edition of the TNM classification for lung cancer. J Thorac Oncol. 2016;11(1):39‐51. 26762738 10.1016/j.jtho.2015.09.009

[4] Al Zreibi C , Gibault L , Fabre E , et al. Surgery for small‐cell lung cancer. Rev Mal Respir. 2021;38(8):840‐847. 34099357 10.1016/j.rmr.2021.05.008

[5] Hanahan D , Weinberg RA . Hallmarks of cancer: the next generation. Cell. 2011;144(5):646‐674. 21376230 10.1016/j.cell.2011.02.013

[6] Xu K , Han H , Luo Y , et al. The angiotensin‐converting enzyme inhibitory state promotes the transformation of non‐small cell lung cancer blood supply pattern toward vasculogenic mimicry formation. Front Oncol. 2021;11:663671. 34221978 10.3389/fonc.2021.663671 PMC8242235

[7] Zhao D , Zheng S , Wang X , et al. iASPP is essential for HIF‐1α stabilization to promote angiogenesis and glycolysis via attenuating VHL‐mediated protein degradation. Oncogene. 2022;41(13):1944‐1958. 35169254 10.1038/s41388-022-02234-9

[8] Li J , Tuo Z , Zong Y , Liu J . Succinate dehydrogenase 5 regulates lung cancer metastasis by reprogramming glucose metabolism. J Thorac Dis. 2021;13(11):6427‐6438. 34992822 10.21037/jtd-21-1769 PMC8662473

[9] Ma Y , Liu Y , Jiang Z . CircRNAs: a new perspective of biomarkers in the nervous system. Biomed Pharmacother. 2020;128:110251. 32480219 10.1016/j.biopha.2020.110251

[10] Liang WC , Wong CW , Liang PP , et al. Translation of the circular RNA circβ‐catenin promotes liver cancer cell growth through activation of the Wnt pathway. Genome Biol. 2019;20(1):84. 31027518 10.1186/s13059-019-1685-4 PMC6486691

[11] Gao G , Wang L , Li C . Circ_0006790 carried by bone marrow mesenchymal stem cell‐derived exosomes regulates S100A11 DNA methylation through binding to CBX7 in pancreatic ductal adenocarcinoma. Am J Cancer Res. 2022;12(5):1934‐1959. 35693076 PMC9185628

[12] Liu T , Song Z , Gai Y . Circular RNA circ_0001649 acts as a prognostic biomarker and inhibits NSCLC progression via sponging miR‐331‐3p and miR‐338‐5p. Biochem Biophys Res Commun. 2018;503(3):1503‐1509. 30029881 10.1016/j.bbrc.2018.07.070

[13] Chen J , Xu S , Chen S , et al. CircPUM1 promotes the malignant behavior of lung adenocarcinoma by regulating miR‐326. Biochem Biophys Res Commun. 2019;508(3):844‐849. 30528736 10.1016/j.bbrc.2018.11.176

[14] Li M , Wang Q , Zhang X , Yan N , Li X . CircPUM1 promotes cell growth and glycolysis in NSCLC via up‐regulating METTL3 expression through miR‐590‐5p. Cell Cycle. 2021;20(13):1279‐1294. 34097560 10.1080/15384101.2021.1934625 PMC8331035

[15] Qi Y , Zhang B , Wang J , Yao M . Upregulation of circular RNA hsa_circ_0007534 predicts unfavorable prognosis for NSCLC and exerts oncogenic properties in vitro and in vivo. Gene. 2018;676:79‐85. 30017736 10.1016/j.gene.2018.07.028

[16] Shangguan H , Feng H , Lv D , Wang J , Tian T , Wang X . Circular RNA circSLC25A16 contributes to the glycolysis of non‐small‐cell lung cancer through epigenetic modification. Cell Death Dis. 2020;11(6):437. 32513983 10.1038/s41419-020-2635-5 PMC7280231

[17] Li J , Zhu Z , Li S , Han Z , Meng F , Wei L . Circ_0089823 reinforces malignant behaviors of non‐small cell lung cancer by acting as a sponge for microRNAs targeting SOX4(). Neoplasia. 2021;23(9):887‐897. 34311177 10.1016/j.neo.2021.06.011 PMC8326602

[18] Zhou R , Ni W , Qin C , et al. A functional loop between YTH domain family protein YTHDF3 mediated m(6)a modification and phosphofructokinase PFKL in glycolysis of hepatocellular carcinoma. J Exp Clin Cancer Res. 2022;41(1):334. 36471428 10.1186/s13046-022-02538-4 PMC9724358

[19] Ge L , Tan W , Li G , Gong N , Zhou L . Circ_0026134 promotes NSCLC progression by the miR‐3619‐5p/CHAF1B axis. Thorac Cancer. 2022;13(4):582‐592. 34985193 10.1111/1759-7714.14301 PMC8841691

[20] Tang J , Li X , Zhao L , Hui J , Ding N . Circ_0006220 contributes to NSCLC progression through miR‐342‐3p/GOT2 Axis. Ann Thorac Cardiovasc Surg. 2022;29:11‐22. 36575008 10.5761/atcs.oa.22-00090 PMC9939679

[21] Fan Y , Wang Q , Shi M , et al. Circ_0020123 promotes NSCLC tumorigenesis via up‐regulating KIAA1522 expression through miR‐940. Cell Cycle. 2022;21(9):894‐907. 35196193 10.1080/15384101.2022.2034093 PMC9037485

[22] Lu Q , Yin H , Deng Y , et al. circDHTKD1 promotes lymphatic metastasis of bladder cancer by upregulating CXCL5. Cell Death Dis. 2022;8(1):243. 10.1038/s41420-022-01037-x PMC9065127 35504887

[23] Wu Z , He X , Chen S . Oncogenic circDHTKD1 promotes tumor growth and metastasis of oral squamous cell carcinoma in vitro and in vivo via upregulating miR‐326‐mediated GAB1. Braz J Med Biol Res. 2021;54(10):e10837. 34287578 10.1590/1414-431X2020e10837 PMC8289343

[24] Castello A , Frese CK , Fischer B , et al. Identification of RNA‐binding domains of RNA‐binding proteins in cultured cells on a system‐wide scale with RBDmap. Nat Protoc. 2017;12(12):2447‐2464. 29095441 10.1038/nprot.2017.106

[25] Zang J , Lu D , Xu A . The interaction of circRNAs and RNA binding proteins: an important part of circRNA maintenance and function. J Neurosci Res. 2020;98(1):87‐97. 30575990 10.1002/jnr.24356

[26] Chan CC , Dostie J , Diem MD , et al. eIF4A3 is a novel component of the exon junction complex. RNA. 2004;10(2):200‐209. 14730019 10.1261/rna.5230104 PMC1370532

[27] Zheng X , Huang M , Xing L , et al. The circRNA circSEPT9 mediated by E2F1 and EIF4A3 facilitates the carcinogenesis and development of triple‐negative breast cancer. Mol Cancer. 2020;19(1):73. 32264877 10.1186/s12943-020-01183-9 PMC7137343

[28] Tang W , Wang D , Shao L , et al. LINC00680 and TTN‐AS1 stabilized by EIF4A3 promoted malignant biological behaviors of glioblastoma cells. Mol Ther Nucleic Acids. 2020;19:905‐921. 32000032 10.1016/j.omtn.2019.10.043 PMC7063483

[29] Yang H , Yang W , Dai W , Ma Y , Zhang G . LINC00667 promotes the proliferation, migration, and pathological angiogenesis in non‐small cell lung cancer through stabilizing VEGFA by EIF4A3. Cell Biol Int. 2020;44(8):1671‐1680. 32281700 10.1002/cbin.11361

[30] Wu Z , Wu J , Zhao Q , Fu S , Jin J . Emerging roles of aerobic glycolysis in breast cancer. Clin Transl Oncol. 2020;22(5):631‐646. 31359335 10.1007/s12094-019-02187-8

[31] Li Z , Zhang H . Reprogramming of glucose, fatty acid and amino acid metabolism for cancer progression. Cell Mol Life Sci. 2016;73(2):377‐392. 26499846 10.1007/s00018-015-2070-4 PMC11108301

[32] Webb BA , Forouhar F , Szu FE , Seetharaman J , Tong L , Barber DL . Structures of human phosphofructokinase‐1 and atomic basis of cancer‐associated mutations. Nature. 2015;523(7558):111‐114. 25985179 10.1038/nature14405 PMC4510984

[33] Webb BA , Dosey AM , Wittmann T , Kollman JM , Barber DL . The glycolytic enzyme phosphofructokinase‐1 assembles into filaments. J Cell Biol. 2017;216(8):2305‐2313. 28646105 10.1083/jcb.201701084 PMC5551713

[34] Lin S , Li Y , Wang D , et al. Fascin promotes lung cancer growth and metastasis by enhancing glycolysis and PFKFB3 expression. Cancer Lett. 2021;518:230‐242. 34303764 10.1016/j.canlet.2021.07.025 PMC8355190

[35] Chowdhury N , Vhora I , Patel K , Doddapaneni R , Mondal A , Singh M . Liposomes co‐loaded with 6‐phosphofructo‐2‐kinase/fructose‐2, 6‐biphosphatase 3 (PFKFB3) shRNA plasmid and docetaxel for the treatment of non‐small cell lung cancer. Pharm Res. 2017;34(11):2371‐2384. 28875330 10.1007/s11095-017-2244-x PMC5754003

[36] Hou P , Meng S , Li M , et al. LINC00460/DHX9/IGF2BP2 complex promotes colorectal cancer proliferation and metastasis by mediating HMGA1 mRNA stability depending on m6A modification. J Exp Clin Cancer Res. 2021;40(1):52. 33526059 10.1186/s13046-021-01857-2 PMC7851923

[37] Li B , Zhu L , Lu C , et al. circNDUFB2 inhibits non‐small cell lung cancer progression via destabilizing IGF2BPs and activating anti‐tumor immunity. Nat Commun. 2021;12(1):295. 33436560 10.1038/s41467-020-20527-z PMC7804955

[38] Jiang X , Guo S , Wang S , et al. EIF4A3‐induced circARHGAP29 promotes aerobic glycolysis in docetaxel‐resistant prostate cancer through IGF2BP2/c‐Myc/LDHA signaling. Cancer Res. 2022;82(5):831‐845. 34965937 10.1158/0008-5472.CAN-21-2988

